# Emergence of *Candida auris* in Brazil in a COVID-19 Intensive Care Unit

**DOI:** 10.3390/jof7030220

**Published:** 2021-03-17

**Authors:** João N. de Almeida, Elaine C. Francisco, Ferry Hagen, Igor B. Brandão, Felicidade M. Pereira, Pedro H. Presta Dias, Magda M. de Miranda Costa, Regiane T. de Souza Jordão, Theun de Groot, Arnaldo L. Colombo

**Affiliations:** 1 Laboratório Especial de Micologia, Disciplina de Infectologia, Universidade Federal de São Paulo, São Paulo 04039-032, Brazil; joao.nobrega@hc.fm.usp.br (J.N.d.A.J.); elaineperol@yahoo.com.br (E.C.F.); 2 Divisão de Laboratório Central, Hospital das Clínicas da Universidade de São Paulo, São Paulo 01246-100, Brazil; 3 Instituto de Medicina Tropical, Universidade de São Paulo, São Paulo 01246-100, Brazil; 4 Westerdijk Fungal Biodiversity Institute, 3508AD Utrecht, The Netherlands; f.hagen@westerdijkinstitute.nl; 5 Department of Medical Microbiology, University Medical Center Utrecht, 3584CS Utrecht, The Netherlands; 6 Comissão de Controle de Infecção Hospitalar, Hospital de Bahia, Salvador 41810-011, Brazil; igorbrasilbrandao@gmail.com; 7 Laboratório Central de Saúde Pública Professor Gonçalo Muniz, Salvador 40295-010, Brazil; felicidade.pereira@saude.ba.gov.br; 8 Centro de Informações Estratégicas de Vigilância em Saúde, Salvador 40279-120, Brazil; pedropresta@hotmail.com; 9 Agência Nacional de Vigilância Sanitária, Brasília 71205-050, Brazil; magda.miranda@anvisa.gov.br; 10 Coordenação Geral de Laboratórios de Saúde Pública, Ministério da Saúde, Brasília 70058-900, Brazil; regiane.jordao@saude.gov.br; 11 Department of Medical Microbiology and Infectious Diseases, Canisius Wilhelmina Hospital (CWZ), 6532SZ Nijmegen, The Netherlands; theundegroot7@gmail.com

**Keywords:** *Candida auris*, COVID-19, intensive care, Brazil

## Abstract

In December 2020, *Candida auris* emerged in Brazil in the city of Salvador. The first two *C. auris* colonized patients were in the same COVID-19 intensive care unit. Antifungal susceptibility testing showed low minimal inhibitory concentrations of 1 µg/mL, 2 µg/mL, 0.03 µg/L, and 0.06 µg/mL for amphotericin B, fluconazole, voriconazole, and anidulafungin, respectively. Microsatellite typing revealed that the strains are clonal and belong to the South Asian clade *C. auris*. The travel restrictions during the COVID-19 pandemic and the absence of travel history among the colonized patients lead to the hypothesis that this species was introduced several months before the recognition of the first case and/or emerged locally in the coastline Salvador area.

## 1. Introduction

After the emergence of *Candida auris* in Venezuela [1], the Pan American Health Organization recommended to the Member States that early detection and notification were necessary to enable the implementation of appropriate measures to prevent and control spread in communities and health care services in the Americas [2]. In March 2017, a task force headed by the Brazilian National Sanitary Surveillance Agency (NSSA) released a document to warn and provide guidance to clinical laboratories and hospital infection control teams (HICT) regarding the emergence of *Candida auris* [3]. The NSSA document recommended the notification and shipment of any strain with phenotypical characteristics of *C. auris* to regional reference laboratories for definitive species identification by MALDI-TOF mass spectrometry and/or by ITS rDNA sequence analysis [3,4].

It was only in December 2020 that the first cultures from patients hospitalized in a tertiary 500-bed hospital from the coastline city of Salvador (Bahia State, Brazil; 12.9777° S, 38.5016° W; ~3 million inhabitants) showed the growth of yeasts identified as *C. auris.*

## 2. Patients and Methods

Case one was a 59-year-old male patient with a previous history of deep-seated venous thrombosis controlled with rivaroxaban 20 mg once daily who was hospitalized 20 October 2020 due to severe acute respiratory syndrome by SARS-CoV2. He had always lived in the Salvador area, without a history of recent international travel. After 43 days under intensive care support (38 days in a COVID-19 intensive care unit) requiring 37 days of mechanical ventilation and hemodialysis for 32 days, blood, central venous catheter tip (CVCt), tracheal aspirate, and urine cultures were requested to investigate a possible infection episode. Cultures returned negative, except for the CVCt culture that grew yeast colonies initially identified as *C. auris* by Vitek 2 automated system (bioMérieux, Marcy-l’Étoile, France). The hospitalization was marked by prolonged corticosteroid therapy (34 days) and several episodes of superinfections by multidrug-resistant Gram-negative bacilli and *Enterococcus faecalis*; empirical anidulafungin therapy had been prescribed from D11 to D28. The patient remained clinically stable, and fever subsided despite no additional antimicrobial therapy. He was discharged on D49.

Case two was a 74-year-old female patient with a previous history of chronic renal insufficiency and dialysis, diabetes mellitus, and hypertension, who was hospitalized on 13 November 2020 due to severe acute respiratory syndrome by SARS-CoV2. She also lived in the Salvador area, with no history of recent international travel. After having spent 30 days at the COVID-19 ICU, having received corticosteroids and noninvasive ventilation support, and still under broad-spectrum antibiotic therapy (meropenem and teicoplanin) due to previous sepsis episodes, blood cultures were requested on 22 December 2020 to investigate a superinfection. Blood cultures returned positive for carbapenem-resistant *Acinetobacter baumannii* and yeast identified as *C. auris* by Vitek 2 (bioMérieux). The hemodialysis catheter exit site at the right jugular vein showed inflammatory signs and was removed. Colistin and anidulafungin were started on 24 December 2020, and control blood cultures collected after 48 h returned negative. The patient had subsequent episodes of catheter-related bloodstream infections by carbapenem-resistant *Morganella morganii* and *Klebsiella pneumoniae* and succumbed on 21 January 2021.

To confirm species identification, MALDI-TOF mass spectrometry (Vitek MS, IVD v3.2, BioMérieux) and ITS rDNA sequencing data analysis were performed [4]. Antifungal susceptibility testing (AST) by broth microdilution reference method of amphotericin B (Sigma-Aldrich, Saint Louis, MO, U.S.), fluconazole (Sigma-Aldrich), voriconazole (Sigma-Aldrich), and anidulafungin (Sigma-Aldrich) was also carried out. To investigate the clonality between the two strains and the genomic relatedness with strains belonging to different clades (South Asian, East Asian, South African, South American, Iran), microsatellite typing with four multiplex PCR reactions was (M2, M3-I, M3-II, M9) performed as previously described [5]. *ERG11* and *FKS1* mutations were also investigated by PCR [6,7] and amplicon Sanger DNA sequencing. Sequence alignment and protein translation were performed with MEGA v10 software [8].

## 3. Results

**Figure 1:**
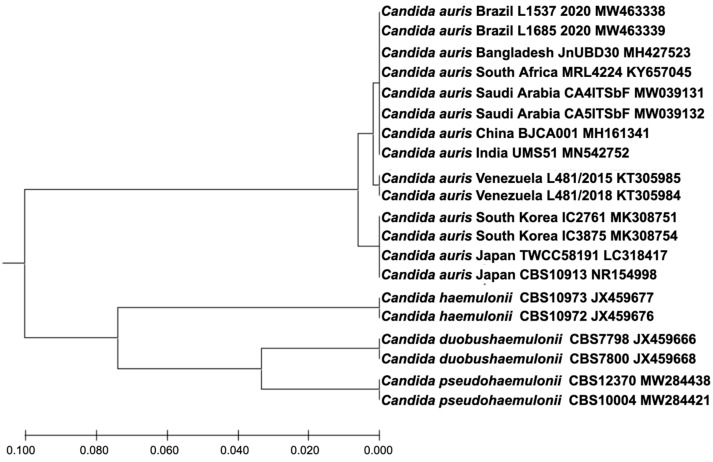
UPGMA phylogenetic tree generated with MEGA X software [8]. There were 20 nucleotide sequences and 299 nucleotide positions in the final dataset. The evolutionary distances were computed using the Maximum Composite Likelihood method and are in the units of number of base substitutions per site.

MALDI-TOF mass spectrometry provided identification of *C. auris* with a 99.9% confidence level. ITS rDNA sequences confirmed species identification as *C. auris* with 100% identity (GenBank accession number LR595924). A phylogenetic tree with *C. auris* and related species (*Candida haemulonii, Candida duobushaemulonii,* and *Candida pseudohaemulonii*) ITS sequences retrieved from GenBank show the clustering of the Brazilian strains (GenBank accession numbers MW463338 and MW463339) with the species *C. auris* (Figure 1).

The AST of the two strains showed minimal inhibitory concentrations of 1 µg/mL, 2 µg/mL, 0.03 µg/L, and 0.06 µg/mL for amphotericin B, fluconazole, voriconazole, and anidulafungin, respectively.

**Figure 2:**
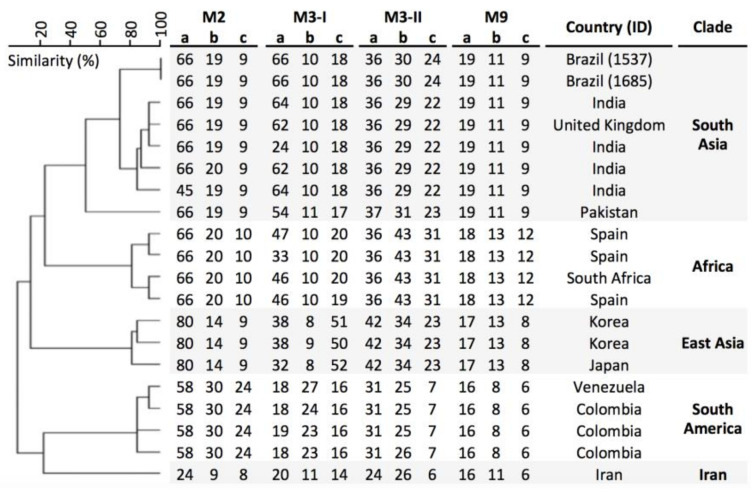
Short tandem repeat (STR) typing of *C. auris* isolates from Brazil. UPGMA dendrogram of the Brazilian (L1537/2020 and L1685/2020) and representative strains from South Asian and other four clades are shown. The scale in the upper left corner represents similarity (%).

The microsatellite analyses revealed that the Brazilian strains were clonal and closely related to the South Asian clade (Figure 2). Despite being related to the South Asian clade, the Brazilian strains showed distinct alleles for the markers M3-I a, M3-II b, and M3-II b (Figure 2).

**Figure 3:**
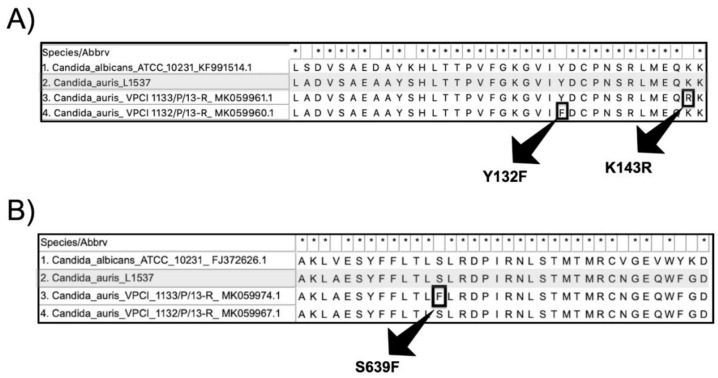
(**A**) Erg11p alignment showing the conserved region between the sites 119 and 145 including the azole wildtype *Candida albicans* ATCC 10231 (1), the Brazilian *Candida auris* strain L1537 (2), the strain VCPI 1133/P/13-R harboring the mutation K143R (3), and the strain VCPI 1132/P/13-R harboring the mutation Y132F. The azole resistance-related amino acid substitutions K143R and Y132F are highlighted with black squares; (**B**) Fks1p alignment showing the conserved region between the sites 627 and 662 including the echinocandin wildtype *Candida albicans* ATCC 10231 (1), the Brazilian *Candida auris* strain L1537 (2), the strain VCPI 1133/P/13-R harboring the mutation S639F (3), and the echinocandin wildtype strain VCPI 1132/P/13-R.

The *ERG11* (GenBank accession number MW619634) and *FKS1* (GenBank accession number MW654481) DNA sequences were translated into the Erg11p and FKS1p, respectively. The azole resistance-related mutations Y132F and K143R, and the echinocandin resistance-related mutation S639F were absent in the Brazilian *C. auris* clone (Figure 3).

## 4. Discussion

When the first case of *C. auris* emerged in Salvador, the epidemiological investigation and the recommended infection prevention and control measures [3] were set in place by the Brazilian Health Ministry Agency and by the local HICT, including environmental and surveillance culture screenings. Until 31 December 2020, nine other patients were found to be colonized. Preliminary epidemiological investigation reveals that none of the *C. auris*-colonized patients had a history of travel outside Brazil, all were from Bahia State, and all were previously admitted to the same COVID-19 intensive care unit. Complete results of the ongoing investigation will be reported in the future.

The emergence of *C. auris* in Brazil has some peculiarities. First, differently from Venezuela and Colombia, the strains are phylogenetically related to the South Asian clade (or clade I), globally the most widespread molecular type. Therefore, this is the first report of local-acquired clade I *C. auris* in Latin America. Second, the Brazilian strains showed some specific microsatellite alleles, and compared to previous reported *C. auris* short tandem repeat profiles [5], these strains would be classified as a unique genotype inside the South Asian clade. Third, unlike most of the strains belonging to the South Asian clade, we found unexpected low antifungal MICs, and the absence of *ERG11* azole resistance-related mutations Y132F and K143R. In China, the first reported clinical isolate of *C. auris* also had low azoles MICs [9]. We hypothesize that these azole wildtype *C. auris* isolates, not yet sufficiently stressed to become resistant, were recently introduced at the hospital. In favor of that hypothesis, the Vitek 2 system of the hospital in Salvador was upgraded in April 2020 and was able to detect *C. auris* from clinical samples. Last but not least, we failed to trace the origin and introduction of *C. auris* clade I in Brazil. The travel restrictions during the COVID-19 pandemic and the absence of travel history among the colonized patients lead to the hypothesis that this species was introduced several months before the recognition of the first case and/or emerged locally in the coastline Salvador area. These findings may be helpful to elucidate the niche and the origin of this pathogen. Indeed, our report corroborates the recent emergence of *C. auris* in COVID-19 intensive care units from other countries [10,11,12]. We also hypothesize that previous healthy *C. auris* colonized individuals that develop severe COVID-19 end up hospitalized in intensive care units, were extensively exposed to antibiotics and invasive medical procedures, and develop superinfections. Thus, the COVID-19 pandemic may be accelerating the introduction and/or spread of *C. auris* in previous *C. auris*-free hospital environments.

The prevalence of *FKS1* mutations in *C. auris* is still low (0–9%), regardless of the clade analyzed [13]. As expected, the Brazilian clone showed high susceptibility to anidulafungin and no *FKS1* mutation was found. Therefore, echinocandins remain a safe first-line therapy for *C. auris* candidemia in our region.

The superinfections by *C. auris* in critically ill COVID-19 patients have been related to high 30-day mortality rates, usually above 50% [10,12,14]. The possible dissemination of *C. auris* in Brazil amid the COVID-19 pandemic could lead to a negative impact on the outcome of these patients. The rising number of severe COVID-19 patients caused by the ongoing second wave is overwhelming the Brazilian hospitals. In a near future, over-occupancy and limited resources for infection control practices, such as prolonged usage of personnel protective equipment due to shortages, will be a fertile ground for *C. auris* to spread, colonize invasive devices and deflagrate healthcare-associated infections.

## Data Availability

The data presented in this study are openly available in GenBank, accession numbers MW463338, MW463339, MW619634, and MW654481.
